# Antiviral Properties of the NSAID Drug Naproxen Targeting the Nucleoprotein of SARS-CoV-2 Coronavirus

**DOI:** 10.3390/molecules26092593

**Published:** 2021-04-29

**Authors:** Olivier Terrier, Sébastien Dilly, Andrés Pizzorno, Dominika Chalupska, Jana Humpolickova, Evžen Bouřa, Francis Berenbaum, Stéphane Quideau, Bruno Lina, Bruno Fève, Frédéric Adnet, Michèle Sabbah, Manuel Rosa-Calatrava, Vincent Maréchal, Julien Henri, Anny Slama-Schwok

**Affiliations:** 1 CIRI, Centre International de Recherche en Infectiologie, (Team VirPath), Univ Lyon, Inserm, U1111, Université Claude Bernard Lyon 1, CNRS, UMR5308, ENS de Lyon, F-69007 Lyon, France; olivier.terrier@univ-lyon1.fr (O.T.); mario-andres.pizzorno@univ-lyon1.fr (A.P.); bruno.lina@univ-lyon1.fr (B.L.); manuel.rosa-calatrava@univ-lyon1.fr (M.R.-C.); 2 Cancer Biology and Therapeutics Team, INSERM, UMR_S 938, Centre de Recherche Saint-Antoine, Sorbonne Universitè, F-75012 Paris, France; Sebastien.Dilly@gmail.com (S.D.); michele.sabbah@inserm.fr (M.S.); vincent.marechal@sorbonne-universite.fr (V.M.); 3 Institute of Organic Chemistry and Biochemistry of the Czech Academy of Sciences, 11720 Prague, Czech Republic; chalupska@uochb.cas.cz (D.C.); humpolickova@uochb.cas.cz (J.H.); evzen.boura@uochb.cas.cz (E.B.); 4 INSERM, UMR_S 938, Centre de Recherche Saint-Antoine, Sorbonne University, and Department of Rheumatology, AP-HP Saint-Antoine Hospital, F-75012 Paris, France; francis.berenbaum@aphp.fr; 5 ISM, UMR-CNRS 5255, Université de Bordeaux, F-33405 Talence, France; stephane.quideau@u-bordeaux.fr; 6 Institut Universitaire de France, F-75231 Paris, France; 7 Genetic and acquired lipodystrophies Team, INSERM, UMR_S 938, Centre de Recherche Saint-Antoine, Sorbonne Université, F-75012 Paris, France; Bruno.Feve@inserm.fr; 8 Department of Endocrinology, AP-HP Saint-Antoine Hospital, Institut Hospitalo-Universitaire de Cardio-métabolisme et Nutrition (ICAN), CRMR PRISIS, F-75012 Paris, France; 9 Service d’Urgences–SAMU–SMUR, Hôpital Avicenne, AP-HP, F-93000 Bobigny, France; frederic.adnet@aphp.fr; 10 Laboratoire de Biologie Computationnelle et Quantitative, Institut de Biologie Paris-Seine, UMR-CNRS 7238, Sorbonne Université, F-75005 Paris, France; julien.henri@sorbonne-universite.fr

**Keywords:** antiviral, drug repurposing, SARS-CoV-2, influenza, structure-based drug design, inflammation, nucleoprotein, oligomerization

## Abstract

There is an urgent need for specific antiviral treatments directed against SARS-CoV-2 to prevent the most severe forms of COVID-19. By drug repurposing, affordable therapeutics could be supplied worldwide in the present pandemic context. Targeting the nucleoprotein N of the SARS-CoV-2 coronavirus could be a strategy to impede viral replication and possibly other essential functions associated with viral N. The antiviral properties of naproxen, a non-steroidal anti-inflammatory drug (NSAID) that was previously demonstrated to be active against Influenza A virus, were evaluated against SARS-CoV-2. Intrinsic fluorescence spectroscopy, fluorescence anisotropy, and dynamic light scattering assays demonstrated naproxen binding to the nucleoprotein of SARS-Cov-2 as predicted by molecular modeling. Naproxen impeded recombinant N oligomerization and inhibited viral replication in infected cells. In VeroE6 cells and reconstituted human primary respiratory epithelium models of SARS-CoV-2 infection, naproxen specifically inhibited viral replication and protected the bronchial epithelia against SARS-CoV-2-induced damage. No inhibition of viral replication was observed with paracetamol or the COX-2 inhibitor celecoxib. Thus, among the NSAID tested, only naproxen combined antiviral and anti-inflammatory properties. Naproxen addition to the standard of care could be beneficial in a clinical setting, as tested in an ongoing clinical study.

## 1. Introduction

The current pandemic of novel coronavirus disease 2019 (COVID-19) was caused by the severe acute respiratory syndrome coronavirus 2 (SARS-CoV-2), the origin of which is not yet known, with first reported cases in Wuhan, Hubei province, China, in December 2019 [1,2]. As of 22 April 2020, there have been 2,475,723 confirmed cases of COVID-19, including 169,151 deaths in 213 countries, which increased to 122,822,505 confirmed cases and 2,709,041 deaths in 223 countries as reported to the WHO on 22 March 2021 (https://www.who.int/emergencies/diseases/novel-coronavirus-2019). SARS-CoV-2 is a beta-coronavirus closely related to the severe acute respiratory syndrome coronavirus-1 (SARS-CoV-1) and the Middle East respiratory syndrome coronavirus (MERS-CoV), which emerged in 2003 and 2012, respectively. These viruses were also transmitted from animals to humans and caused severe respiratory diseases in afflicted individuals.

Patients with severe COVID-19 suffer from a cytokine storm due to an uncontrolled immune response, endothelial dysfunction leading to thrombosis and hypoxia, which, ultimately, may lead to multiorgan failure and sepsis. The exacerbated inflammatory response in severe COVID-19 cases presents similarity to the cytokine storm observed in severe cases of H5N1 Influenza virus infection and overall inflammation, as in the 1918 Influenza A pandemic [3]. In that regard, corticoid treatments, such as dexamethasone, have resulted in a lower mortality among patients with severe symptoms [4]. We hypothesize that drugs combining anti-inflammatory and antiviral effects could even more efficiently reduce the symptoms of respiratory distress and inflammation caused by COVID-19 [5]. 

Viral infection activates a cyclooxygenase-2 (COX-2) inflammatory cascade that is most marked in the initial inflammatory phase [6]. SARS-CoV-2 infection also upregulates COX-2 in human cell culture and mouse models [7]. The effectiveness of COX-1/COX-2 inhibition by non-steroidal anti-inflammatory drugs (NSAIDs) such as naproxen and celecoxib, discouraging the inflammasome activation could limit the cytokine storm [8]. Although it was initially suggested that NSAIDs could increase ACE2 receptor expression, thereby possibly promoting viral entry, a recent clinical trial attested the safety of COX-2 inhibitors and suggested their ability to reduce IL6 levels [9]. NSAID treatment by two commonly prescribed drugs, ibuprofen and meloxicam, had no effect on ACE2 expression, viral entry, or viral replication but may influence COVID-19 outcomes by dampening the inflammatory response and production of protective antibodies [7]. Recent reports suggested that among COVID-19 patients requiring hospitalization, individuals treated with ibuprofen or naproxen were more commonly not requiring ventilation [10]. 

Our previous work showed that naproxen, an approved anti-inflammatory drug, is an inhibitor of both COX and of Influenza A virus nucleoprotein [11]. Naproxen binding to the Influenza A virus nucleoprotein blocked viral RNA association with the nucleoprotein and impeded the nucleoprotein self-association [11,12]; consequently, naproxen strongly reduced viral transcription/replication in infected cells and protected mice against an infection with Influenza A virus [11,13].

Viral nucleoproteins (N) are unique to the virus and no equivalent of these proteins are found in the host cell. N bind to genomic RNA to form a virion core comprising a ribonucleoprotein (RNP) complex that assumes a long helical structure. The RNP is required for replication and transcription, in which the N protein plays a critical role. In SARS-CoV-2, interactions between N and the non-structural protein-3 are also important for replication. Through its interaction with the coronavirus membrane (M) protein, the N protein drives virus assembly and budding. In addition to its role in different aspects of the viral cycle, the N protein not only highjacks cellular processes, including the progression of the host cell cycle and apoptosis, but also modulates the immune response, notably through its ability to block the interferon response [14]. N represses host antiviral response (as RNA interference and RIG-I mediated interferon) [15], N targets the stress granule protein G3BP1, an essential antiviral protein which is known to induce innate immune response [16]. N function is often regulated by phosphorylation [17,18]; indeed, mutations were observed in residues of the serine-rich domain. Additionally, the N of SARS-CoV-1 virus was shown to directly bind to the COX-2 promoter to regulate its expression [19]. Altogether, N are attractive targets for potential antivirals with anti-inflammatory properties [20,21].

In the present pandemic context, we aim at impeding N association with viral RNA and induced N oligomerization with repurposed drugs [22]. We therefore target the N-terminal domain (NTD) of SARS-CoV-2 nucleoprotein, known to bind RNA and explore the repurposed NSAID naproxen, its binding to NTD and its prevention of N oligomerization, thereby its antiviral activity against SARS-CoV-2 pandemic virus, as previously implemented for the Influenza A virus [12,13,23]. Naproxen, being an easily affordable drug, could help in the worldwide, long-lasting pandemic crisis we have already been experiencing for a year.

## 2. Results

### 2.1. Structure-Based Modeling of Naproxen Binding to the Nucleoprotein of SARS-CoV-2

**Figure 1:**
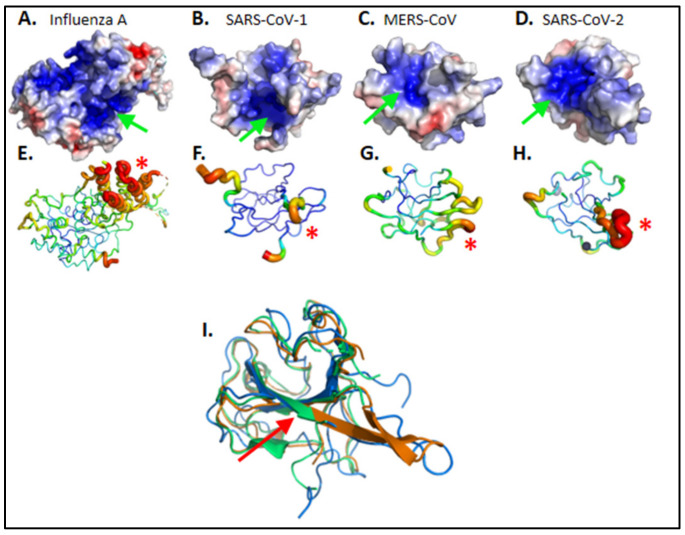
**Structural comparison of viral nucleoproteins N.** (**A**) Influenza A 2IQH.pdb and of amino-terminal domains of (**B**) SARS-CoV-1 1SSK.pdb (**C**) MERS-CoV 6KL2.pdb (**D**) SARS-CoV-2 6VYO.pdb. Electrostatic surface potentials are computed according to Adaptive Poisson-Boltzmann Solver Electrostatics plugin from PyMOL in standard parameters. Electronegative potentials are colored red while electropositive potentials are colored blue. A large electropositive cleft is visible on each protein, pointed with a green arrow; it maps the putative RNA interaction sites. (**E**–**H**) Main chains flexibilities are approximated as crystallographic b-factors, with main chain ribbon diameter proportional to b-factor value; red is highest b-factor while blue is lowest b-factor. Most mobile elements are identified by a red asterisk (*); they group on one external side of the protein, facing solvent. Mobile elements of highest b-factors are proximal to electropositive surfaces, supporting an interaction scenario where (1) RNA docks onto an electro-complementary surface before (2) the protein conformation is induced into the N-RNA complex of highest stability. (**I**) Pairwise structural alignment of SARS-CoV-1 (blue), MERS-CoV (green) and SARS-CoV-2 (orange) nucleoproteins. The red arrow emphasizes the conserved RWYFYY sequence. Images were ray traced with PyMOL version 2.0.6. Orientations were selected to highlight discussed properties.

The nucleoproteins N of enveloped, positive-sense, single-stranded viruses Coronavirus (CoV) share with negative-sense single-stranded viruses such as Influenza A virus the ability to bind to and protect genomic viral RNA without sequence specificity and to form self-associated oligomers [20,21,23,24]. Despite the limited sequence similarity between them, the N-terminal domains (NTD) of the three coronaviruses considered here, SARS- Cov-1, MERS- CoV, SARS-CoV-2 and Influenza A virus, all presented a wide, positively charged groove in which the viral negatively charged RNA binds, Figure 1A,D [21,25,26,27]. The coronavirus N proteins present a central antiparallel sheet of 3–5 β-strands structure and flexible linkers and loops, with emphasis on the large extrusion carrying many basic residues, which likely helps adjusting to RNA binding [28]. The C-terminal domain is also structured and involved in interactions with viral RNA and dimerization. N also contains intrinsically disordered linkers interacting with the M protein. Further associations of N, viral RNA and replication complex (nsp12 and cofactors nsp7, nsp8) into RNP remained yet poorly understood. As we aimed to impede N association with viral RNA and induced oligomerization, we restricted ourselves to the N amino-terminal domain (NTD) for identification of ligands, with or without COX inhibition, targeting this domain as potential antivirals.

CoV N NTD presented flexible loops/linkers evaluated here from the crystallographic b factor (Figure 1E–H) as previously identified in Influenza A nucleoprotein [29]. N NTD well-structured central β-sheet concentrated most of the aromatic residues (Figure 1I): SARS-CoV-2 nucleoprotein NTD counts 14 aromatic residues among a total of 124 modeled in 6VYO.pdb. The consecutive, 5-aromatics pentapeptide ^108^WYFYY^112^ is located at the center of the electropositive groove (Figure 1I, red arrow). This contrasts with the nucleoprotein of the Influenza A virus, which has a single aromatic residue (Y148) within its RNA binding groove in which naproxen was shown to bind [11]. As naproxen usually forms hydrophobic/stacking interactions with hydrophobic or aromatic residues of the protein, we expected from these observations that the N-terminal domain of CoVs N may offer more than a single binding site for naproxen.

**Figure 2:**
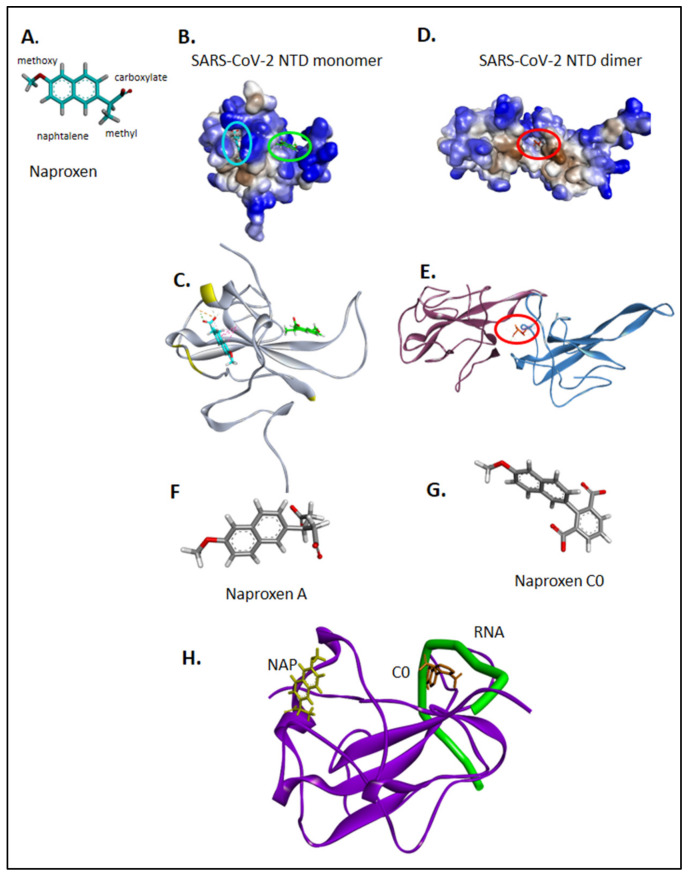
Binding sites of naproxen to N NTD monomer and dimer; comparison with naproxen derivatives. Naproxen is shown in (**A**), with details on its functional moieties as discussed in the text. Panels (**B**,**C**) show the binding sites of naproxen on monomeric N NTD (PDB 7ACT) [28], the main (more frequent) binding site represented in cyan, is associated with the dimeric interface, highlighted in (**C**) in yellow; the minor site of naproxen on monomeric N NTD is represented in green. In panels (**D**,**E**) is shown the main binding site of naproxen on dimeric N NTD (PDB 6VYO) [27]. For details, see also Supplementary Figure S2. The structures of naproxen A and C0 are shown in panels (**F**) and (**G**), respectively. Panel (**H**) summarizes the RNA binding site (shown in green) deduced from RMN studies [28], the main binding site of naproxen on the left and the main binding site of naproxen C0, located within the RNA binding groove. (See also Table 1 and Supplementary Figure S2).

Indeed, we identified two binding sites for naproxen onto the two solved NTD structures, the monomeric structure of SARS-CoV-2 nucleoprotein NTD solved by NMR spectroscopy [28] (PDB 7ACT) and in the dimer of dimer crystal structure PDB 6VYO [25]. In the most frequent monomeric binding site shown in light blue in Figure 2A–C, the naphthalene aromatic core of naproxen stacked on W52, while its carboxylate made electrostatic interactions with R149 and its methyl group formed hydrophobic interactions with I146. This site is located in the vicinity of T76, N77, S79 and H145 (highlighted in yellow in Figure 2C), residues of monomer A interacting with monomer D in the X-ray structures of N NTD crystallized as a dimer of dimer [30]. Consistently, in the structure PDB 6VYO, naproxen main binding site was stabilized by hydrophobic with W52, I157, L104, Y112 and electrostatic interactions with R92 and R107 of the conserved sequence R^107^WYFYY^112^ at the dimeric interface (Figure 2D,E). Naproxen was most stable in this site, with a ΔG = −37 ± 1 Kcal/mol, an RMSD of 2.9 ± 1.2 Å (Table 1 and Supplementary Figure S1). The second most frequent binding site of naproxen on the NTD monomer is shown in green in Figure 2B,C, where naproxen interacted with R92, R93 and Y109, Y111, residues stabilizing the association of RNA with N NTD or AMP [28] (Figure 2F). This second site for naproxen binding onto the monomer was consistently found in the dimeric structure (Table 1). Supplementary Figure S2 summarizes the interactions of naproxen with dimeric SARS-CoV-1 and monomeric MERS-CoV. Accordingly, the following order of free binding energies of naproxen binding to the N-terminal domain of N were calculated: SARS-CoV-1 (ΔG = −34 ± 4 Kcal/mol) ≈ MERS-CoV (ΔG = −32 ± 3 Kcal/mol) ≈ SARS-Cov-2 (ΔG = −37 ± 1 and −32 ± 2 Kcal/mol for site 1 and 2, respectively). This comparison suggested naproxen binding to all three tested N NTD CoV proteins.

We previously developed naproxen derivatives, called naproxen A and naproxen C0 (Figure 2F,G) that both lacked the ability to inhibit COX [12]. Naproxen C0 was simultaneously more potent against Influenza A virus in its antiviral effect (lower IC50) and less toxic (higher CC50) than naproxen [12,13]; naproxen A had no antiviral effect. Interestingly, naproxen C0 also bound to monomeric N of SARS-CoV-2 (Table 1). Naproxen C0 mainly bound at a site associated with RNA binding, in contrast with naproxen, which mainly bound to a site involved in N oligomerization, although both compounds presented similar scores (Figure 2H). Naproxen A binding to monomeric or dimeric N NTD was less favorable than naproxen binding (Table 1). Taken together, the data suggested that naproxen and its derivatives bound to N NTD in the order naproxen ≥ naproxen C0 > naproxen A.

We also compared naproxen with the pain killer acetaminophen (paracetamol), which bound very weakly to N dimer, while celecoxib, a COX-2 inhibitor, stabilized the dimeric interface and bound monomeric N even tighter than naproxen did.

### 2.2. Evidence for In Vitro Binding of Naproxen to Recombinant N-Terminal Domain of SARS-CoV-2 N

The N-terminal domain of the nucleoprotein of SARS-CoV-2 (residues 50–173) with an N-terminal His6 tag was expressed in *E. coli* as detailed in the Experimental section and in Supplementary Figure S3. FPLC analysis identified the NTD protein purified as a dimer (30 KDa) using anion exchange to remove host nucleic acids. Moreover, the same expressed NTD protein purified by removal of nucleic acids contaminants by enzymatic means [24] formed a monomer. NTD was reported to be purified as a dimer [31] while a recent SEC-MALS analysis reported the NTD purified as a monomer [32].

#### 2.2.1. Fluorescence Assay

**Figure 3:**
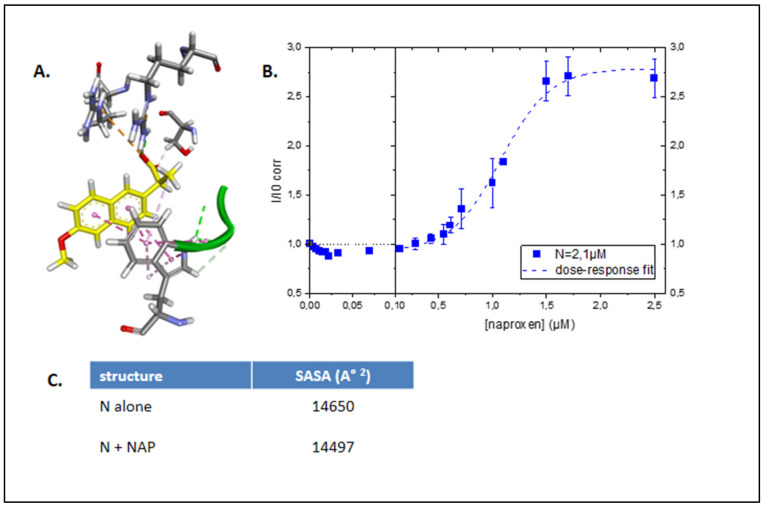
Naproxen binds to recombinant N-terminal domain of SARS-CoV-2 N. (**A**): Close-up view of naproxen interacting with W52 (PDB 6VYO). (**B**): Normalized intrinsic fluorescence of N, corrected for the inner filter effect, as a function of naproxen concentration using 2.1 µM N in 20 mM Tris buffer pH = 7.9, 100 mM NaCl. (**C**): evaluation of the solvent-accessible surface area in the N dimer without and with naproxen.

Dimeric NTD was used in the fluorescence assay. As naproxen interacted with aromatic residues of N dimer, in particular W52 (Figure 3A), we reasoned that this binding would modify the intrinsic protein fluorescence (excitation 280 nm, emission 327 nm–337 nm). At these wavelengths, the contribution of naproxen fluorescence was minimal (less than 10% of the signal) but naproxen contributed to the total absorbance at 280 nm, and masked the intrinsic absorbance of N, a process called inner filter effect. The data were corrected for the inner filter effect due to naproxen absorption at 280 nm. Figure 3B shows that indeed the fluorescence increased upon addition of naproxen, with an apparent EC50 = 1.1 ± 0.1 µM. We anticipated an increase in the intrinsic fluorescence upon naproxen binding by the calculation of the solvent-accessible surface area (SASA) in N alone that decreased in the presence of naproxen, in agreement with aromatic residues as W52 being in a more buried environment due to naproxen binding (Figure 3C). Taken together, these data confirmed that naproxen bound to dimeric N. In SARS-CoV-2.

#### 2.2.2. Thermal Shift Assay Monitored by Dynamic Light Scattering (DLS)

Further experiments were performed to test whether naproxen would stabilize the nucleoprotein against heat-induced denaturation by thermal shift assay (TSA). Usually, TSA are monitored by fluorescence; here we used dynamic light scattering (DLS) to avoid any perturbation of the measurements by direct interactions between the ligands and the fluorescent probe. DLS is a useful technique for monitoring protein size and their apparent thermal stability. The N NTD monomer alone in buffer was characterized by an apparent hydrodynamic diameter Dh = 5.3 ± 0.3 nm for 80% of the signal together with a larger form Dh = 450 ± 100 nm. Upon heating, the size of N NTD increased to about 5000 nm, with a transition, the apparent melting temperature Tm at 51 ± 1 °C (Table 2). When naproxen bound to N NTD, the Tm increased to 56 ± 1°C, consistent with naproxen stabilizing N by 5 °C (Table 2). The same experiment performed with the analgesic compound paracetamol or with naproxen A did not stabilize N NTD either (Table 2).

#### 2.2.3. Competition on N NTD Binding of RNA and the Ligands Evidenced by Fluorescence Anisotropy

**Figure 4:**
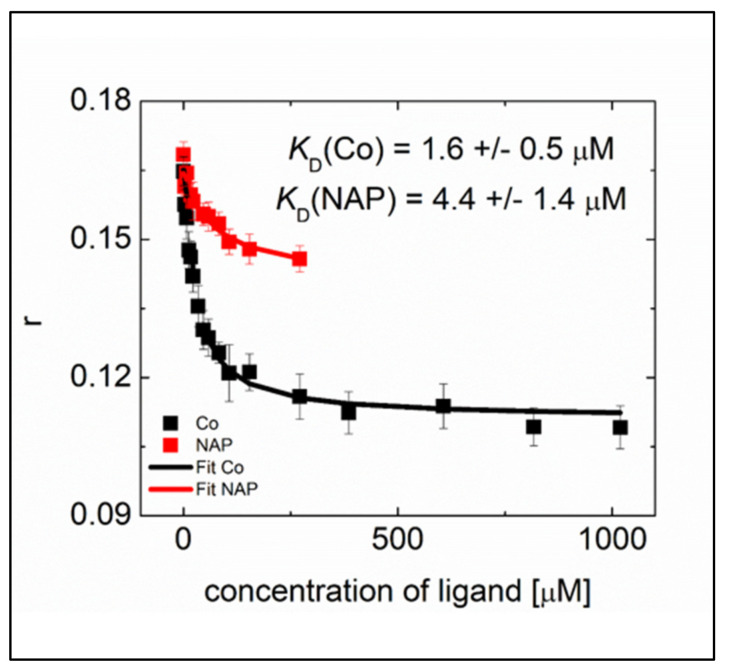
Competition on N NTD of binding of RNA with naproxen or naproxen C0 deduced from fluorescence anisotropy r.

We previously used an RNA binding assays to gain a deeper insight into the interaction of N NTD with RNA [29]. We titrated hexachlorofluorescein labeled ssRNA using the N-NTD domain and monitored the increase in fluorescence anisotropy. The RNA binding assay revealed that the wild type N-NTD binds the 10-mer RNA with a K_D_ of 8.3 ± 0.8 μM. Here, we determined the displacement by the naproxen or naproxen C0 ligands of RNA from the SARS-CoV-2 N-NTD (see also the experimental section). This competing interaction decreased the anisotropy signal of the N NTD—RNA complex upon the addition of naproxen (up to 271 μM) or its derivative naproxen C0 (up to 1018 μM) (Figure 4). The data could be fitted by a competition between the ligands and RNA for binding to N NTD (1/1 interaction). The extent of anisotropy decrease revealed a more extensive competition of naproxen C0 with RNA than that observed with naproxen. Interestingly, naproxen C0 was more efficient than naproxen in the RNA displacement, as deduced from the determination of their respective K_D_s: K_D_ (naproxen C0) = 1.6 ± 0.5 µM and K_D_ (naproxen) = 4.4 ± 1.4 µM. These data are consistent with distinct main binding sites of the two ligands deduced from modeling studies (Figure 2H), where naproxen C0 occupied a site within the RNA binding site while naproxen was mainly located at the dimer interface. Therefore, these differences in binding sites suggest that naproxen may interfere with N oligomerization induced by RNA binding and naproxen C0 would not. The below experiments test this hypothesis.

#### 2.2.4. N Oligomerization Assay Monitored by DLS in the Presence of Nucleic Acids and the Effect of Various Ligands Thereof

**Figure 5:**
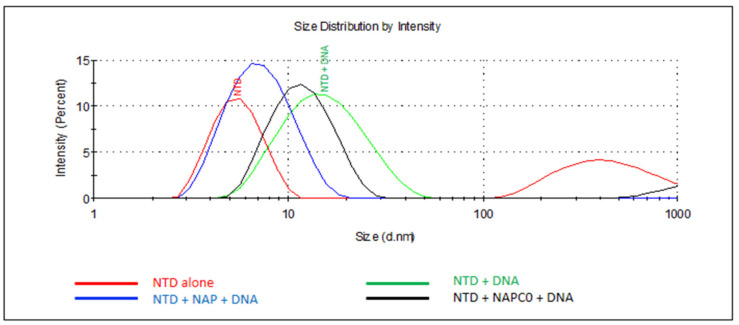
Naproxen competes with DNA-induced N oligomerization. Size distribution of N NTD alone (red) or in the presence of 48 mer DNA (green), demonstrating the DNA-induced oligomerization of N NTD. Naproxen (blue) addition to N largely impeded N NTD oligomerization while naproxen C0 (black) only somewhat decreased the size of the N-DNA complex. The sizes are described in the text. T = 20 °C, [NTD] = 60 µM, [48mer DNA] = 29.4 µM and naproxen or naproxen C0 were used at 37.5 µM. In these conditions, naproxen A or acetaminophen could not impede N oligomerization (Table 2).

Upon the addition of a single-stranded DNA fragment of 48 nucleotides long at a ratio of one DNA per two NTD monomers, Dh almost instantaneously increased to Dh = 15.7 ± 0.7 nm, attesting to the formation of oligomers (Figure 5 and Table 2). The same experiment was performed with added naproxen, or the analgesic drug acetaminophen (paracetamol) at a stoichiometry of one ligand per two NTD monomers. While acetaminophen did not modify NTD oligomerization in the presence of 48-mer DNA (Table 2), naproxen drastically reduced the size of the DNA–NTD complex to Dh = 7.9 ± 0.3 nm. Naproxen C0 only slightly reduced the size of the DNA–NTD complex to Dh = 12.7 ± 0.7 nm as compared to Dh = 15.7 ± 0.7 nm without a ligand. The addition of naproxen A did not modify the size of the oligomers observed without a ligand (Dh = 16.0 ± 0.8 nm). Taken together, N NTD monomers formed oligomers in the presence of 48-mer DNA, an oligomerization process only described for N C terminal domain [33]. Moreover, NTD oligomerization was modified by N binders in the decreasing order: naproxen >> naproxen C0, with naproxen A and acetaminophen being non-inhibitory. Interestingly, the COX-2 selective inhibitor celecoxib mainly enhanced the formation of large species, presumably aggregates.

Competing with N binding to RNA and modifying N oligomerization would repress replication and decrease the level of RNA produced by the virus. The experimental quantification of the viral RNA by RT-qPCR tests this hypothesis in the below antiviral assays.

### 2.3. Naproxen Inhibits SARS-CoV-2 Infection in Vero E6 Cells and in Reconstituted Human Airway Epithelia (HAE)

**Figure 6:**
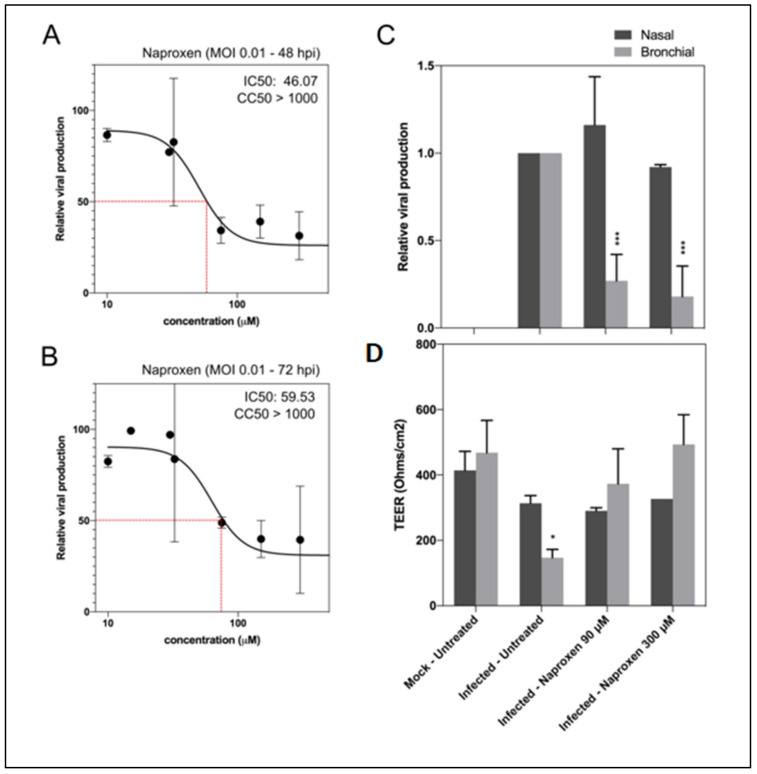
Naproxen inhibits SARS-CoV-2 infection in Vero E6 cells and in HAE. (**A**,**B**) Dose–response curves of naproxen at 48 and 72 hpi in infected Vero E6 cells. Cells were infected at MOI 0.01 with SARS-CoV-2 and then treated 1 h postinfection with a large range of concentration of naproxen; (**C**) Relative intracellular viral genome quantification and (**D**) trans-epithelial resistance (TEER in Ohms/cm^2^) between the apical and basal poles in nasal and bronchial HAE at 48 hpi. Results are expressed in relative viral production compared to the infected untreated control and relative TEER compared to t = 0 (before infection). *** *p* < 0.001 and * *p* < 0.05 compared to the infected untreated (viral titers) or uninfected (TEER) groups by Student’s *t*-test. Data are representative of three independent experiment.

Based on the docking analysis and the biophysical experiments described above, we then evaluated the potential antiviral activity of naproxen against SARS-CoV-2 in vitro. As shown in Figure 6A,B, naproxen efficiently reduces viral replication in African green monkey Vero E6 cells in a dose-dependent manner. A unique treatment at 1 h postinfection (hpi) resulted in 50% inhibitory concentration (IC50) values of 46.07 µM at 48 hpi and 59.53 µM at 72 hpi. We determined a cytotoxic concentration CC50 > 1000 µM. This value compares well with the CC50 previously determined in MDCK and A549 cells to be 1605 ± 300 µM [11]. The selectivity index (SI) was SI = CC50/IC50, SI > 21.71 and 19.79, respectively.

We further evaluated the antiviral effect of naproxen using a more biologically relevant experimental model, notably the nasal and bronchial MucilAirTM reconstituted human airway epithelia (HAE) [33]. Developed from biopsies of nasal or bronchial cells differentiated in the air/liquid interphase, these models reproduce with high fidelity most of the main structural, functional and innate immune features of the human respiratory epithelium that play a central role in the early stages of infection and constitute robust surrogates to study airway disease mechanisms and for drug discovery [33]. Postinfection treatment of nasal HAE with 90 µM or 300 µM naproxen did not show an antiviral effect at 48 hpi compared to the mock-treated control (Figure 6C).

Conversely, significant reductions in intracellular SARS-CoV-2 viral titers were observed for the two treatment conditions in bronchial HAE (73% and 82% reduction versus mock-treated control, respectively). This reduction in viral titers correlated with naproxen inducing a protective effect of the bronchial epithelium integrity, as shown by trans-epithelial electrical resistance (TEER), considered as a surrogate of epithelium integrity. The TEER values in both naproxen groups were comparable to those of the uninfected and untreated control (Figure 6D) and significantly higher than those of the untreated control (Figure 6D). The antiviral effects of naproxen in reconstituted human bronchial epithelium were consistent with the IC50 value determined in Vero E6 cells. However, the lack of antiviral effect of naproxen on nasal epithelium was puzzling. The viral load is usually lower in the nasal cavity as compared to the lungs [34,35]. The nasal epithelium is an important portal for initial infection, and may serve as a key reservoir for viral spread across the respiratory mucosa and an important locus mediating viral transmission. The number of host cells as pneumocytes and alveolar macrophages permissive to viral entry is higher in the lungs than the mucous cells in the nasal cavity. Moreover, recent interactome analysis supports the hypothesis that SARS-CoV-2 preferentially hijacks host proteins available in the lung tissue, and N targets stress granule protein G3BP1, an essential antiviral protein which is known to induce innate immune response [36].

Interestingly, we previously determined that the peak of viral replication was reached earlier in bronchial (48–72 hpi) than in nasal HAE, in which a progressive increase in infectious viral titers was observed until at least 96 hpi [33]. Using this model, this differential antiviral effect between airway sites was observed for naproxen in this work and for remdesivir in a recent report that indicates an antiviral effect was mainly observed in the lower respiratory tract of non-human primates [33]. It is interesting to note that naproxen was found to be very protective against viral-induced damages at 48 h postinfection in the reconstituted bronchial epithelium (Figures 4 and 6D in [33]). Therefore, we speculate that the lack of naproxen effect in nasal epithelium as opposed to its antiviral effect in pulmonary epithelium could be associated with the differential expression or activity of host factor(s), and/or with slower replication kinetics in the nasal versus pulmonary epithelia.

**Figure 7:**
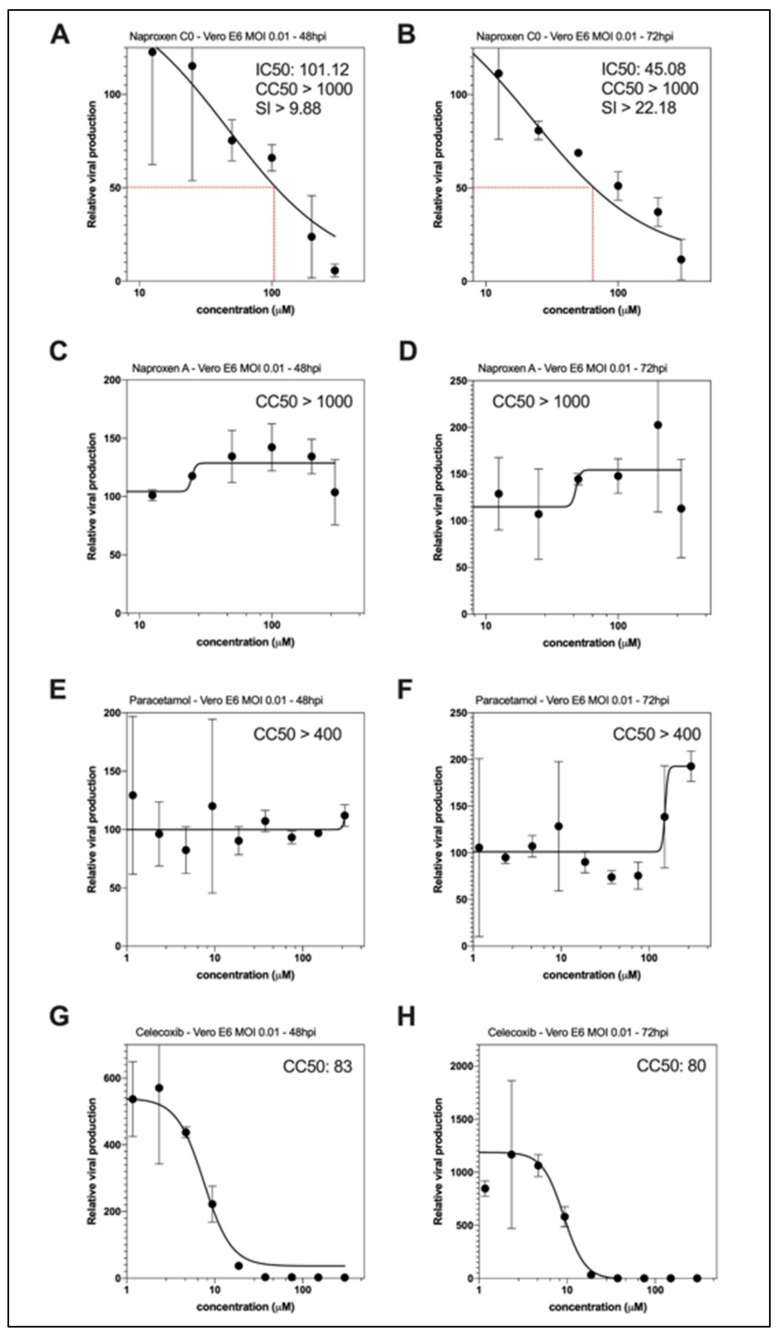
Antiviral effect of naproxen C0, as compared to the naproxen A derivative, acetaminophen and the COX2 inhibitor celecoxib in infected Vero E6 cells. Dose–response curves of naproxen C0 (**A**,**B**) or naproxen A (**C**,**D**) at 48 and 72 hpi in infected Vero E6 cells. Using the same experimental protocol as in Figure 4, Vero E6 cells were infected at MOI 0.01 with SARS-CoV-2 and then treated 1h postinfection with a large range of concentration of naproxen C0 or A or paracetamol or celecoxib. Panels (**E**,**F**) and (**G**,**H**) show the lack effect of acetaminophen (paracetamol) and the mainly proviral effect of celecoxib (COX2 inhibitor) on viral replication, demonstrating the specificity of naproxen among the COX inhibitors tested (Table 2).

As naproxen is both a COX and N inhibitor, we further tested whether the host-dependent anti-inflammatory property of naproxen via COX inhibition is required for the observed antiviral effect. The naproxen derivatives we previously developed were lacking an inhibition of COX [12,13]; thereby, it is suitable to test whether they would also repress SARS-CoV-2 replication by the inhibition of N. Accordingly, a unique treatment at 1 hpi with naproxen C0 results in IC50 values of 101.12 and 45.08 µM at 48 and 72 hpi, respectively, with comparable CC50 values > 1000 µM (Figure 7A,B). These results indicated that naproxen C0 still presented antiviral properties while it did not inhibit COX [13], very likely by the inhibition of N and subsequent decrease on viral replication. In contrast, naproxen A did not reduce viral replication (Figure 7C,D). Naproxen was further compared with the pain killer acetaminophen (paracetamol) and the specific COX2 inhibitor celecoxib in their ability to inhibit viral replication (Figure 7E,F and G,H, respectively). While paracetamol was safe, mainly not affecting viral replication, celecoxib did not present a clear antiviral effect out of its toxicity limit, CC50, being 80 µM.

## 3. Discussion

We demonstrate here that the ability of naproxen to bind the nucleoprotein of SARS-CoV-2 characterized by an IC50 of 1.1 µM to dimeric NTD and stabilize N NTD against heat denaturation. Naproxen competed with RNA binding to N NTD, yielding a K_D_ = 4.4 ± 1.4 µM using a monomeric NTD, and drastically decreased N oligomerization as shown by a combination of modeling, fluorescence anisotropy, thermal shift assay and DLS data. Consequently, naproxen inhibited viral replication in both the VeroE6 cell line and in a model of reconstituted pulmonary epithelium with an IC50 of 50 ± 10 µM and a CC50 > 1000 µM. COX inhibition was not necessary involved in naproxen inhibition of viral replication as suggested by the antiviral effect of naproxen C0, although its effect was somewhat lower than that of naproxen IC50 = 75 ± 25 µM (Table 2).

The experimental data supported the different binding sites of naproxen and naproxen C0 on N NTD proposed by modeling studies. Naproxen C0 mainly bound in the RNA binding groove at a site associated with: (i) a more efficient competition of naproxen C0 with RNA for binding to N NTD as compared to naproxen, as deduced from fluorescence anisotropy data; (ii) consequently, naproxen C0 had a lower ability of inhibiting N NTD oligomerization than naproxen did, since these two sites are on different faces of the protein; (iii) an overall weaker antiviral effect of naproxen C0 than that of naproxen. Naproxen A could not inhibit N oligomerization, yet had no antiviral effect. Both hydrophobic and electrostatic interactions stabilized naproxen in an area of N NTD involved in protein–protein interactions, with minimal steric hindrance for naproxen (Figure 2H). In contrast, naproxen C0 could experience some steric effects likely arising from the two aromatic rings, and less favorable electrostatic interactions of its two carboxylate moieties that disfavor binding at this site; naproxen C0 better fitted at an alternative site at the highly charged and wider base of the RNA-binding loop located at the edge of the β-sheet.

Our data likely link the inhibition of N oligomerization with viral replication inhibition. Indeed, the pain reliever acetaminophen (paracetamol), which very weekly bound to N deduced from modeling and DLS data, did not modify N oligomerization and had no antiviral effect [37]. COX inhibitors [38] such as ibuprofen and meloxicam did not affect SARS-CoV-2 entry or replication in vitro [7]. The proviral effect of celecoxib, a specific COX-2 inhibitor, further supported the hypothesis that an enhancement of N oligomerization/aggregation was associated with its proviral effect at low concentration, without clear antiviral effect in its safety concentration range.

Naproxen’s mechanism of action demonstrated here involved the inhibition of N oligomerization associated with its antiviral effect. Nevertheless, we cannot rule out that naproxen may bind other viral targets, such as the viral 3C-like protease, as suggested from modeling studies and experimental determination of an IC50 of 3.45 µM [39]. Indeed, during replication and viral particle assembly, viral proteases cleave polyproteins expressed by the virus to produce a number of essential Non Structural Proteins (NSPs); hence, protease-inhibitors are a popular class of antiviral candidates. Nevertheless, as N is one of the most abundant structural proteins in CoVs [33], SARS-CoV-1 virions containing multiple copies of the N protein (ca 1000 copies), we assume that impeding N function associated with viral replication would be the main mode of action explaining the observed antiviral effect of naproxen.

Broad-spectrum antiviral activity of naproxen: In addition to the antiviral activity against SARS-CoV-2 virus demonstrated here, naproxen also exhibited antiviral properties in cellular and rodent models of Influenza A and B [11,13,40] and the IC50 of naproxen for SARS-Cov-2 compares well to IC50 = (25 ± 7) µM and SI = 64 of naproxen determined for Influenza A at the same MOI = 10^−2^. Naproxen also inhibited the replication of the unrelated Zika virus [41]. Therefore, from three unrelated single-stranded RNA virus, naproxen presented broad-spectrum antiviral properties. Moreover, the combination of clarithromycin, naproxen and oseltamivir reduced the mortality of patients hospitalized for H3N2 Influenza infection when compared to oseltamivir alone in a phase IIB/III clinical trial [42].

Taken together, we demonstrate in this study that among the NSAIDs tested, only naproxen has direct antiviral activity on SARS-CoV-2 replication and, importantly, protected the pulmonary epithelium from the pandemic virus-induced damage, thus combining antiviral and anti-inflammatory effects. These very encouraging results prompted us to test in a clinical trial, named ENACOVID, the effect of addition of naproxen to the standard of care in severely ill patients. This trial is supported by APHP, Assistance Publique, Hôpitaux de Paris areas [43] https://www.clinicaltrialsregister.eu/ctr-search/search?query=eudract_number:2020-001301-23 (accessed on 13 April 2021).

## 4. Methods

### 4.1. Modeling

The following X-ray structures extracted from the Protein Data Bank (PDB) have been used: N-terminal domain of SARS-CoV-2: 6VYO [25], 7ACT [28], SARS-CoV-1: 1SSK [26], MERS-CoV: 6KL2/6KL5 [21]; The Influenza A nucleoprotein PDB 2IQH was used as a comparison [44]. The binding mode of naproxen for the protein structures was assessed in a two-step protocol. First, the binding site was defined by blind docking to the whole protein using the docking program AutoDock Vina [45]. The docking space was defined visually in order to encompass the proteic model with a 2 Å margin at least as a tetragonal box. Three replicas with the default scoring function Vina [45] were performed to check the convergence of the experiment. The relevance of the binding sites identified by blind docking was confirmed by the cavity detection algorithm of Discovery Studio version 2020, which detected similar sites. A site-docking was then performed using Libdock with energy minimization using smart minimizer (Discovery Studio version 2020) to more precisely identify the naproxen binding mode. In this docking, three replicas were also applied but on a restricted space, encompassing the cavity where the ligand was blind docked. For each protein, the most representative pose of naproxen (i.e., the pose most often found) was selected. The resulting protein-NP complexes were finally refined by a molecular dynamics simulation using the CHARMm force field [46] and the standard dynamic cascade protocol of Discovery studio version 2020. This protocol started with a first minimization of 1000 steps using the Steepest Descent algorithm and a RMSD gradient of 1 Å, followed by a second minimization of 2000 steps using the Adopted Basis Newton-Raphson algorithm and a RMSD gradient of 0.1 Å. The third step involved heating from 50 °K to 300 °K with a fourth step of equilibration step during 1 ns and a fifth production step. The time of the production step was initially set at 20 ns, but a time extension was applied if the ligand was not stable (See Supplementary Figure S1). Three replicas were carried out for each complex. For each trajectory, the displacement of the ligands was studied by rmsd calculation. The structures with a close position (rmsd < 1 Å) were then grouped in the same cluster. The representative structure (i.e., with the smallest average rmsd from all other structures of the cluster) of the largest cluster of each complex was selected and then used for an estimation of the free binding energy using implicit Distance-dependent dielectrics solvent model found in Discovery version 2020.

### 4.2. Materials

Naproxen was purchased from Sigma, Saint Quentin Fallavier, France (reference N5160). Acetaminophen, Celecoxib and the 24-mer and 48-mer fragments were also purchased from Sigma. The naproxen derivatives were synthesized as previously reported [12]. The N-terminal domain of the nucleoprotein of SARS-CoV-2 (residues 50-173) with an N-terminal His6 tag was cloned in pET-28a vector (pET28-His6-NP-NTD) and used for two independent preparations, hereafter named batch 1 and batch 2. Batch 1 was obtained by heterologous expression at 15 °C for 16 h in *E. coli* BL21 bacterial strain (DE3 (GeneCust, Boynes, France). The recombinant protein (15 KDa) found in the soluble fraction was purified on a Ni^2+^-NTA affinity column and SP sepharose ion exchange chromatography, and presented a single band revealed by SDS PAGE. The yield was 1.5 mg of pure protein per liter of culture. Batch 2 was obtained by heterologous expression at 30 °C for 3 h in *E. coli* Rosetta2 pLysS strain. Cell pellet was resuspended in 20 mM Tris pH = 7.9, 100 mM NaCl (buffer A) supplemented with 1 mg/mL CEW lysozyme, 0.15 mg/mL RNaseA, 200 units of benzonase and 10 mM MgCl_2_, incubated 30 min at room temperature and lysed by sonication as described in [24]. The soluble fraction was submitted to Ni^2+^-affinity chromatography including a 1 M NaCl washing step. Eluate was injected on a preparative Superdex200 size-exclusion chromatography and isocratically eluted in buffer A. Central peak fractions were assembled and ultrafiltered to 51 mg/mL as measured by nanodrop spectrophotometry (extinction coefficient ε = 26,930 M^−1^cm^−1^ and molecular weight 14,636 g/mol). The yield was 19 mg of pure protein per liter of culture. Batch 2 presented an Abs280/Abs260 ratio of 1.8, as expected for a protein solution devoid of nucleic acids.

### 4.3. Size-Exclusion Chromatography

Full-length SARS-CoV-2 nucleocapsid was described as a homodimer in solution [32]. Both batches of recombinant NP-NTD that we purified elute as single peaks over size-exclusion columns (Supplementary Figure S3). However, while batch 1 elution volume is consistent with the apparent molecular weight of a 30 kDa dimer, batch 2 elution volume rather supports a monomeric state with an apparent molecular weight smaller than 17 kDa.

### 4.4. Fluorescence Measurements

Fluorescence spectra were recorded on a PTI Instrument, using excitation and emission slits set at 2.5 nm, equipped with a thermostated cell holder set at 20 °C, λexc = 280 nm, λem = 290–390 nm. The protein samples (purified as a dimer) were usually used at a concentration range of 2–10 µM in a 20 mM Tris buffer at pH = 7.9 containing 100 mM NaCl. The protein concentration was determined at 280 nm using ε = 26,930 M^−1^cm^−1^ while the extinction coefficient of naproxen at 280 nm was 2350 M^−1^cm^−1^.

### 4.5. Fluorescence Anisotropy

The steady-state fluorescence anisotropy experiments were performed using a Fluoromax-4 spectrofluorimeter (Horiba, Scientific), in a thermostated cell holder. The excitation and emission bandpass filters were centered at 538 nm and 553 nm, respectively. For these experiments the N NTD was expressed and purified as before [29]. Titration experiments were performed in 10 mM Tris pH 8.0, 50 mM NaCl, 2 mM β-mercaptoethanol. The mixture of 7.25 μM N NTD and 100 nM fluorescently labeled RNA (UCUCUAAACG labeled with 5′-hexachlorofluorescein, Sigma) was titrated with naproxen or naproxen C0 until the signal of fluorescence anisotropy of N NTD–RNA complex was decreasing (up to 271 μM naproxen and 1018 μM naproxen C0). The anisotropy data were fitted by the homemade script in Matlab (Mathworks, Natick, MA, USA). The steady-state anisotropy is defined as the following ratio of vertical and horizontal emission intensities, IV and IH, respectively, r = (I_v_ − I_H_)/(I_V_ + 2I_H_). The anisotropy r is an intensity weighted average of the contribution of the bound and unbound fraction:

r = ((r_bound_ xq + r_unbound_ *(c_RNA_ − x)))/((xq + c_RNA_ − x)),

where c_RNA_ and x are the total concentration and concentration of bound labelled RNA, respectively. r_bound_ and r_unbound_ are the anisotropies of the bound and free RNA, respectively, and q is the ratio of quantuum yields of the fluorophore in the bound and unbound RNA molecules. x is obtained by solving following chemical equilibria:

K_D RNA_=((c_protein_ − x − y)*(c_RNA_ − x))/x,

K_D ligand_ = ((c_protein_ − x − y)*(c_ligand_ − y))/y.

Here, c_protein_ and c_ligand_ stand for the total protein and non-fluorescent ligand concentration and y is the concentration of bound non-fluorescent ligand. K_D RNA_ and K_D ligand_ are corresponding dissociation constants.

### 4.6. Dynamic Light Scattering Measurements

The experiments were performed on a Malvern nanosizer apparatus. The temperature was set at 20 °C, and 10 scans with a duration of 10 s was determined in duplicate for each time and sample. The size distribution in the intensity of the scattered light was obtained using the Cumulants method from the instrumental software, yielding the hydrodynamic diameter. The N NTD concentration used was in the range of 40–60 µM, in 20 mM Tris buffer at pH = 7.9 containing 100 mM NaCl. The sequence of the 48 mer DNA was: 5′ ATA TAT ATC TAT GTC CAT ATA TAT ATA AAA CAC AGC GTG TGT GTG TAA 3′. The melting experiments were performed at a heating rate of 1 °C/minute over the range 30 °C to 65 °C in sealed disposable cuvettes. The apparent melting temperature, Tm, was determined by the first derivative of the melting curve.

### 4.7. Virus

All experiments involving the manipulation of infectious SARS-CoV-2 were performed in biosafety level 3 (BSL-3) facilities, using appropriate protocols. The BetaCoV/France/IDF0571/2020 SARS-CoV-2 strain used in this study was isolated directly from a patient sample as described elsewhere [34].

### 4.8. Dose–Response Antiviral Evaluation in Vero E6 Cells

Six-well multi-well plates were seeded with VeroE6 cells 24 h before infection (day −1). On day 0, 90%-confluent wells were washed twice with PBS and infected with SARS-CoV-2 at a multiplicity of infection (MOI) of 0.01. One hour after infection, the inoculum was removed and cells were subsequently treated with the indicated dilutions of naproxen in DMEM. DMEM was used as mock-treatment control. Plates were then incubated at 37 °C and 5% CO_2_. Supernatants were collected at 48 and 72 hpi and stored at −80 °C for viral RNA extraction with the QiAmp viral RNA Kit (Qiagen, Courtabeuf, France) and titration by RT-qPCR as described elsewhere [34]. Drug 50% cytotoxic concentration (CC50), 50% inhibitory concentration (IC50) and selectivity index (SI) values were calculated using the Quest Graph IC50 calculator (AAT Bioquest, Euromedex, Souffelweyersheim, France).

### 4.9. Evaluation of Antiviral Activity in Reconstituted Human Airway Epithelia (HAE)

MucilAirTM reconstituted HAE were obtained from Epithelix SARL (Geneva, Switzerland) and maintained in air-liquid interphase with specific culture medium in Costar Transwell inserts (Corning, NY, USA) according to the manufacturer’s instructions. After two washes with warm OptiMEM medium (Gibco, ThermoFisher Scientific, Les Ulis, France), apical poles were inoculated with a 150 μL dilution of virus in OptiMEM medium at a MOI of 0.1. OptiMEM was used as mock-infection control. One hour after viral infection, MucilAirTM culture medium containing or not (untreated) specific dilutions of naproxen were applied through the basolateral poles. Treatments were repeated at 24 hpi. At 48 hpi, HAE cells were harvested in RLT buffer (Qiagen) and total ARN was extracted using the RNeasy Mini Kit (Qiagen) and stored at −80 °C for subsequent titration by RT-qPCR. Variations in transepithelial electrical resistance (Δ TEER) were measured using a dedicated volt-ohm meter (EVOM2, Epithelial Volt/Ohm Meter for TEER) and expressed as Ohm/cm^2^.

## Figures and Tables

**Table 1:** Binding sites on N NTD in dimeric (subunits A and D in PDB 6VYO) or monomeric forms, of naproxen, naproxen derivatives and two NSAIDs-like drugs as deduced from modeling studies.

Compound	N Dimer.PDB 6VYO ΔG (Kcal/mol)/RMSD (Å)	Binding Site	Score Dimer/% Population	N NTD Monomer PDB 7ACT Binding Site	Score Monomer/ % Population
**Naproxen**	−37 ± 1 /2.9 ±1.2	A: W52, I157 D: R92, L104, S105, R107, Y112	−7.9/40%	W52, I146, R149	−6.4/33%
	–32 ± 2 /8 ±4	A: T148, N150 D: R92, I94	−6.4/25%	A50, A90, R92, R93	−5.4/13%
**Naproxen A** ^1^		A: N77, W52, I146 D: T54, R92, R107, Y109	−7.1/35%	A90, R92, R107, Y109	−6.1/15%
**Naproxen C0** ^1^		A: W52, N75, I157 D: R107	−7.9/25%	A90, R92, R107, Y109	−6.3/50%
**Acetaminophen (paracetamol)**		A: H145, I146	−5.7/50%	W52, I146, R149	−5.0/25%
**Celecoxib** ^1^		A: W52, N75, I157 D: A55,R107	−8.1/40%	W52, I146, R149	−7.4 /50%

^1^ Only the main binding site of the ligands onto N NTD is described.

**Table 2:** Summary of the compounds, their COX inhibition, and their ability to bind to N NTD, to modify NTD oligomerization induced by nucleic acid binding and their antiviral properties.

Compound	COX Inhibition	N Binding µM Fluo /Fluo Anisotropy	Tm (°C)/ΔTm (°C) ^4^	Oligomer Inhibition/Enhancement	Oligomer Size (nm)^.5^	Mean IC50 µM	CC50 µM
**None**			51 ± 1		15.7 ± 0.7		
**Naproxen**	COX1 + COX2	1.1 ± 0.1 ^2^/ 4.4 ± 1.4 ^3^	56 ± 1 /+5	+++	7.9 ± 0.3	52 ± 6	>1000
**Naproxen A**	None (ref 11)	Nd	47 ± 2, 58 sh /−4	-	16.0 ± 0.8	No inhibition	>1000
**Naproxen C0**	None (ref 11)	/ 1.6 ± 0.5 ^3^	-	+	12.7 ± 0.7	73 ± 25	>1000
**Acetaminophen** **(paracetamol)**	COX1 + COX2 ? ^1^	Nd	46 ± 2, 54sh /−5	-	14.0 ± 1.0	No inhibition	>400
**Celecoxib**	COX2	Nd	−6	/Enhancement	^7^ Formation of large particles of 3 µm as compared to N + DMSO (12 nm)	proviral	80

Nd: not determined, sh: shoulder in the derivative curve. ^1^: The mechanism of action of paracetamol is still a matter of debate, COX inhibition is not completely demonstrated, hence the question mark. ^2^: N NTD (2 µM) purified as a dimer. ^3^: N NTD (7 µM) purified as a monomer, 100 nM RNA [28]. ^4^: NTD (48 µM) monomer, [compound] = 75 µM, 20 mM Tris Buffer pH = 7.9, 100 mM NaCl, 20 °C. ^5^: NTD (60 µM) monomer, [compound] = 37.5 µM, [48-mer] = 29.4 µM, 20 mM Tris Buffer pH = 7.9, 100 mM NaCl, 20 °C. ^6^: As Celecoxib is soluble in DMSO and not in buffer, it cannot be compared to the other members of the series (all water soluble) in the thermal shift assay. ^7^: In the oligomerization assays, N NTD + DMSO was compared to N NTD + celecoxib in DMSO.

## Data Availability

Preliminary report have been made available on bioRxiv; https://doi.org/10.1101/2020.04.30.069922 (accessed on 13 April 2021). Other data are not available.

## Supplementary Materials

The following are available online, Figure S1: Representative MD trajectories of the naproxen-N complexes of SARS-CoV-2 (dimeric N, sites 1 and 2), SARS-CoV-1 (monomeric N) and MERS-CoV (dimeric N). Figure S2: Binding of Naproxen to N NTD Coronaviruses, Figure S3: Size-exclusion chromatography of SARS-CoV-2 nucleocapsid NTD.

## Sample Availability

Samples of the naproxen derivatives could be available upon request from the authors.
