# Supplementary material for: Antiviral Properties of the NSAID Drug Naproxen Targeting the Nucleoprotein of SARS-CoV-2 Coronavirus
Source: Molecules. 2021 Apr 29;26(9):2593. doi: 10.3390/molecules26092593 (PMC8124269; doi:10.3390/molecules26092593)
Supplement: Supplementary file 1 [file molecules-26-02593-s001.zip › molecules-1192346-SI.pdf]

Antiviral properties of the NSAID drug naproxen

Targeting the nucleoprotein of SARS-CoV-2 Coronavirus

Olivier Terrier[1], Sébastien Dilly[2#], Andrés Pizzorno[1#], Dominika Chalupska[3], Jana Humpolickova[3], Evžen Bouřa[3], Francis Berenbaum[4], Stéphane Quideau[5,6], Bruno Lina[1], Bruno Fève[7,8], Frédéric Adnet[9], Michèle Sabbah[2], Manuel Rosa- Calatrava[1&], Vincent Maréchal[2&], Julien Henri[10], and Anny Slama- Schwok[2*]

Appendix A: Supplementary data

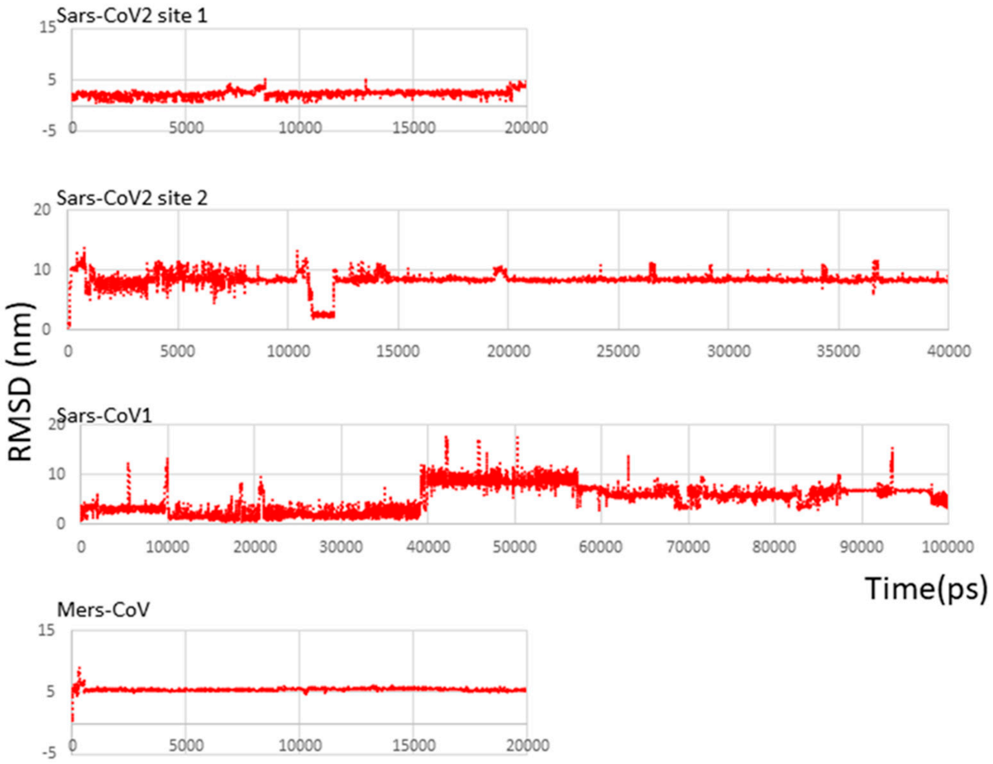

Supplementary Figure 1: Representative MD trajectories of the naproxen-N complexes of SARS-CoV-2 (dimeric N, sites 1 and 2), SARS-COV-1 (monomeric N) and MERS-CoV (dimeric N).

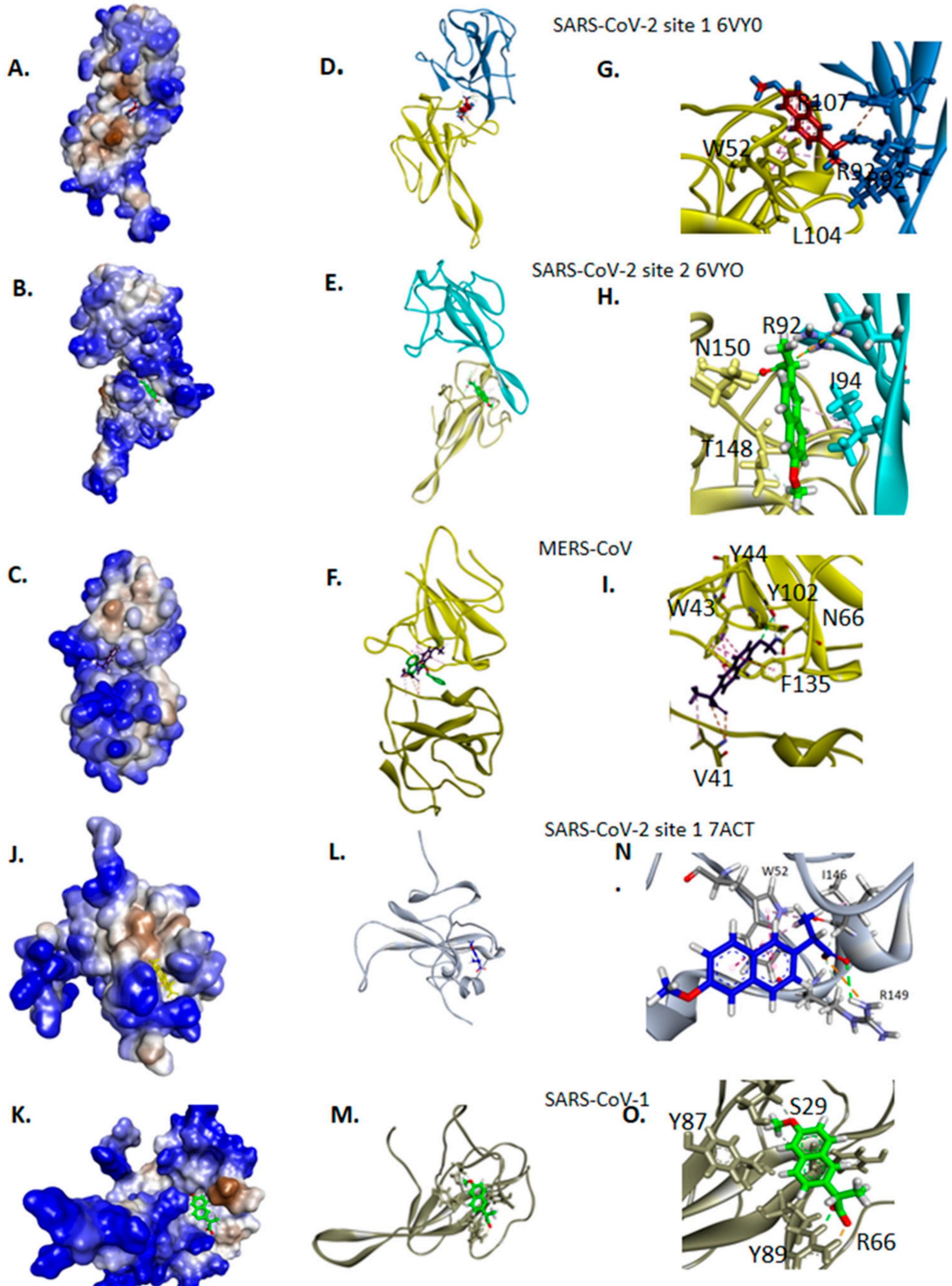

Sup. Figure 2

Supplementary Figure 2: Binding of Naproxen to N NTD Coronaviruses Each structure is represented in hydrophobic (brown) to hydrophilic (blue) surface (first column, in ribbon (second column), a close-up is shown in the third column. Binding of naproxen to the N-terminal domain of SARS-CoV2 site 1 (most frequent site): panels A, D and G: the carboxylate formed salt bridges with R92, R107 and sometimes interacted via by polar interactions with S105, the latter belonging to the sequence $R^{107}WYFYY^{112}$ of monomer D, L104 of monomer D made hydrophobic contacts with the methyl of the methoxy group, the aromatic core of naproxen stacked on W52 and sometimes made hydrophobic contacts with Y112 (of the sequence $R^{107}WYFYY^{112}$). Binding of naproxen to the N-terminal domain of SARS-CoV2 site 2: panels B, E and H: the carboxylate interacted by polar and electrostatic interactions with N150 (monomer A) and R92 (monomer D), respectively, T148 of monomer A made hydrophobic contacts with the methyl of the methoxy group, the aromatic core of naproxen made hydrophobic contacts with I94 or L104 (monomer D). Binding of naproxen to the N-terminal domain of MERS-CoV: panels C,F and I: the carboxylate interacted by polar interactions with N66 or N68 or T134 (monomer D), the methyl group formed hydrophobic contacts with V41 or A109 (monomer A), the aromatic core of naproxen stacked on W43 and F135 (monomer D), Y44 and Y102 of the sequence $R^{97}WYFYY^{102}$ (monomer D) made both hydrophobic interactions and polar interactions (via its OH group) with the methoxy group of naproxen. The ligand crystallized in this structure is depicted in green for comparison. Binding of naproxen to monomeric N-terminal domain of Sars-CoV-2 main

site (PDB 7ACT), panels J.L. and N: the naphthalene aromatic core of naproxen stacked on W52, while its carboxylate made electrostatic interactions with R149 and its methyl group formed hydrophobic interactions with I146. Binding of naproxen to monomeric N-terminal domain of SARS-CoV-1: panels K, M and O: the carboxylate interacted by electrostatic interactions with R66 and sometimes R70, the oxygen of the methoxy group made polar interaction with S29, the aromatic core of naproxen was in hydrophobic/ stacking interactions with surrounding Y87 and Y89 of the sequence $R^{85}WYFYY^{90}$. π-alkyl interactions were also often found with Y87 or Y89. As a comparison, naproxen binding to Influenza A N that involved electrostatic interactions of the carboxylate with R361, a cation -π interaction with R355 and hydrophobic interaction with Y148 and interactions of the last C-terminal F (not shown) with the methyl of the methoxy group.

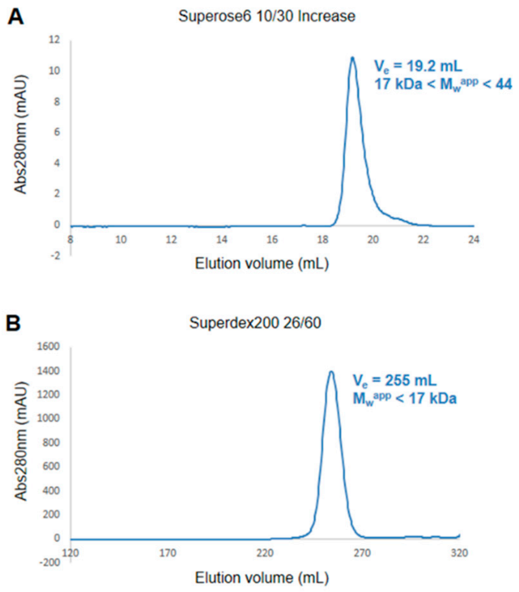

Supplementary Figure 3: Size-exclusion chromatography: Full-length SARS-CoV-2 nucleocapsid was described as a homodimer in solution. Both batches of recombinant NP-NTD that we purified elute as single peaks over size-exclusion columns (figure S3A). However, while batch 1 elution volume is consistent with the apparent molecular weight of a 30 kDa dimer, batch 2 elution volume rather supports a monomeric state with an apparent molecular weight smaller than 17 kDa (Figure S3B).
